# Study on the Introduction of Solid Fat with a High Content of Unsaturated Fatty Acids to Gluten-Free Muffins as a Basis for Designing Food with Higher Health Value

**DOI:** 10.3390/ijms22179220

**Published:** 2021-08-26

**Authors:** Milena Kupiec, Anna Zbikowska, Katarzyna Marciniak-Lukasiak, Katarzyna Zbikowska, Małgorzata Kowalska, Hanna Kowalska, Jarosława Rutkowska

**Affiliations:** 1 Faculty of Food Assessment and Technology, Institute of Food Sciences, Warsaw University of Life Sciences (WULS-SGGW), Nowoursynowska St. 159c, 02-776 Warsaw, Poland; milena_kupiec@sggw.edu.pl (M.K.); anna_zbikowska@sggw.edu.pl (A.Z.); 2 Faculty of Medicine, Medical University of Warsaw, Żwirki i Wigury St. 61, 02-091 Warsaw, Poland; 3 Faculty of Chemical Engineering and Commodity Science, Kazimierz Pulaski University of Technology and Humanities, Chrobrego St. 27, 26-600 Radom, Poland; mkowalska7@vp.pl; 4 Department of Food Engineering and Process Management, Institute of Food Sciences, Warsaw University of Life Sciences (WULS-SGGW), Nowoursynowska St. 159c, 02-776 Warsaw, Poland; hanna_kowalska@sggw.edu.pl; 5 Faculty of Human Nutrition, Institute of Human Nutrition Sciences, Warsaw University of Life Sciences (WULS-SGGW), Nowoursynowska St. 159c, 02-776 Warsaw, Poland; jaroslawa_rutkowska@sggw.edu.pl

**Keywords:** gluten-free product, oleogels, fatty acids, solid fat replacers, quality of lipid fraction, microtomography, texture

## Abstract

Background: Shortenings are high in undesirable nutritionally saturated fatty acids. The aim of the study was to produce gluten-free muffins (GFM) of increased health quality and available to people intolerant to gluten, in which the shortenings were replaced with solid oleogels, consisting of 95% rapeseed oil. Methods: The dough and baked products were subjected to physical, textural, and structural analyses. Moreover, the fatty acids composition, chemical quality of fats extracted from muffins, and color of the products were determined. The dough was also observed at 600× magnification in bright field and polarized light microscopy, and microtomographic analysis of the structure of GTM was performed. Results: There was no effect of the type of lipids on physical properties, including water content in gluten-free muffins. However, the baked products differed in total porosity and brightness, as well as intensity of red and yellow colors. The use of rapeseed oil oleogels, instead of shortening in the muffin recipe, resulted in a decrease in the dietary undesirable SFA in lipid fractions (by approximately 40%), an increase in the content of MUFA (by approximately 30%), and an increase in the content of PUFA (by approximately 15%), with acceptable chemical quality. Conclusions: Research confirms the possibility of obtaining products with increased nutritional value available to consumers on a gluten-free diet.

## 1. Introduction

Already, Hippocrates saw the relationship between medicine and food, claiming that “Let thy food be thy medicine and medicine be thy food” [1]. Food products, regardless of the degree of processing, consist of molecules of various chemical compounds.

Fats (lipids), apart from carbohydrates and proteins, belong to the group of macronutrients provided with the diet. They mainly include triacylglycerol molecules, but also, among others, phospholipids, glycolipids, or cholesterol [2,3]. Fats are a necessary component of the diet as they provide energy, essential fatty acids, and vitamins (A, D, E, K). In addition, they improve the palatability and texture of food and make it easier to swallow a bite [3].

From a technological point of view, an important ingredient of confectionery products is gluten, which is known for its unique viscoelastic properties. Its molecules allow obtaining high-quality products [4,5]. According to Codex Alimentarius, it is a protein fraction of wheat, barley, rye, or oats (or their derivatives) that is insoluble in water and 0.5 M NaCl [6]. However, in practice, the term gluten is used to define a protein network formed with the participation of water molecules as a result of the formation of hydrogen, covalent or disulfide bonds between amino acids [5,7]. Its consumption may cause various gastrointestinal or parenteral ailments among people intolerant to this ingredient [5,8,9]. This is one of the reasons for the growing interest of consumers in the so-called gluten-free food [10] but also in pro-health food [11,12]. An increasing number of people (not only diagnosed with celiac disease) give up eating gluten [11] or limit products with a high content of saturated fatty acids [13].

Traditional bakery and pastry products, e.g., biscuit muffins, contain 25–40% addition of solid fat, e.g., shortening. It is a composition of refined and modified vegetable oils, which, due to the relatively high content of saturated fatty acids (SFA, usually approximately 40%) and the possible presence of *trans* fatty acids (TFA), is not a desirable food ingredient [14,15,16]. A diet rich in SFA and TFA results in consistently increased serum Low-Density Lipoprotein, which may consequently lead to coronary heart disease (CHD) [17]. Due to the negative impact of TFA on the body, the Regulation of the European Commission no. 2019/649 limited the content of industrial TFA to 2g/100g of fat [18]. However, solid lipid (i.e., with TFA and SFA) enables creating an appropriate structure of confectionery products [19,20] and therefore, shortenings producers are replacing TFA with SFA [14,15]. 

It is possible to replace bakery shortenings with an unfavorable dietary composition of fatty acids (FA) with oleogels [16]. These are systems, structured with molecules of gelling substances, consisting of at least 90% vegetable oils (e.g., rapeseed oil—RO) with a nutritious fatty acid profile [21]. Oleic acid chains, which are present in the RO up to 70% [22], by replacing SFA with other molecular species of monounsaturated fatty acids in the diet, lower the risk of heart disease [17]. Substituting shortenings with oleogels in food could positively affect the prevention of overweight and obesity, which, according to the World and Health Organization, 39% of adults struggled with in 2016 [23].

Considering the need to remove molecular species of fat from the diet, which increases the risk of cardiovascular diseases and the growing interest in gluten-free products, this study attempts to replace commercial shortening with rapeseed oil oleogels (with a high content of unsaturated fatty acids) in gluten-free muffins. The influence of such reformulation on the quality parameters of the raw and baked dough as well as on the quality of the lipid fraction of muffins was analyzed.

## 2. Results

### 2.1. Specific Gravity of Muffin Dough

**Figure 1:**
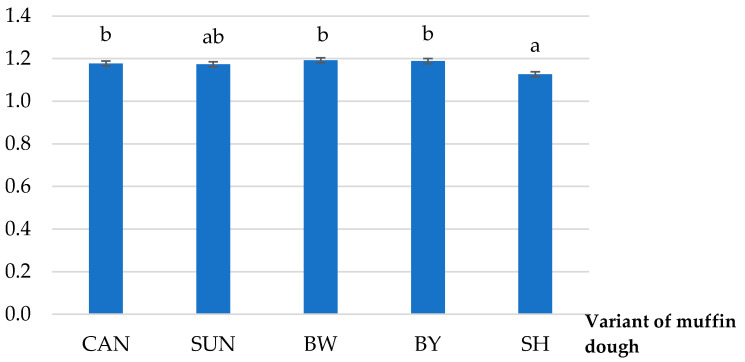
Specific gravity of gluten-free-muffin dough (g/cm^3^), which contained shortening (SH) or oleogel made with the addition of a single wax: candelilla (CAN), sunflower (SUN), beeswax white (BW), or yellow (BY); a, b—different letters above each column indicate statistical difference at α = 0.05.

The beneficial effect of shortening (SH) was confirmed in terms of specific gravity (Figure 1). The SG parameter for the dough with SH had the lowest value—1.13 g/cm^3^, which did not significantly differ from the SUN variant (1.17 g/cm^3^). It can be assumed that it is related to the high melting point of sunflower wax (Table 1). The other raw cakes containing rapeseed oil structured by vegetable or animal waxes did not differ significantly from each other in terms of SG (Figure 1).

### 2.2. Analysis of the Microstructure of Dough at Room Temperature

**Figure 2:**
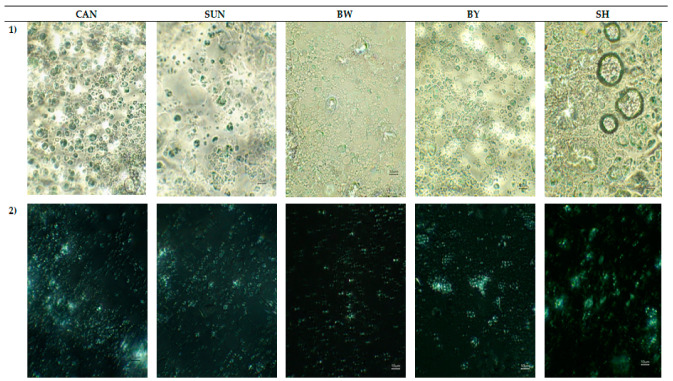
Microstructure of dough of gluten-free muffins, which contained shortening (SH) or oleogel with candelilla (CAN), sunflower (SUN), beeswax white (BW), or beeswax yellow (BY) as the fat component, as observed in bright field (**images at the top—1**) and polarized light (**images at the bottom—2**) in 600× magnification.

The bright field technique (BFT) and polarized light microscopy (PLM) enabled comparing the raw dough microstructure of gluten-free muffins containing shortening or rapeseed oil, which were structured with different plant and bees waxes (Figure 2). Air bubbles (in the form of circles) were observed using BFT in the control variant—with shortening (Figure 2(1)).

The presence of starch grains, also in aggregates, was observed using PLM (Figure 2(2)). The formation of clusters is a characteristic feature of rice starch [24]. Their greatest amounts were seen in the variants with oleogel with beeswax yellow (BY) or shortening (SH). The remaining raw cakes were dominated by single grains smaller than 10 µm, with rounded shapes. Six hundred times magnification of the samples also allowed the observation of birefringence ability of the rice starch molecules with characteristic Maltese crosses (Figure 2(2)).

### 2.3. Physical Parameters of Gluten-Free Muffins 

Bakery goods did not differ significantly in terms of weight, diameter, height, volume, and water content (Table 2).

For statistical analysis, Pearson’s correlation was also performed to determine the effect of air entrainment in dough on the geometrical parameters of the baked products. A high correlation was demonstrated between the height of the muffins and the specific gravity (SG) of the dough (Pearson’s correlation coefficient r was |−0.54|). The higher the SG value, the less airless the dough and the lower the height of the baked muffin. The regression equation was determined: Height = 45.53–10.97·SG. In other analyzed geometric parameters, no significant influence of specific gravity was demonstrated (Table 3).

### 2.4. Texture Profile Analysis

To determine the textural properties of gluten-free muffins, the analysis of hardness, springiness, cohesiveness, and chewiness of the crumb was performed. All tested products did not differ significantly in terms of the mentioned parameters (Table 4). The hardness, expressed by the maximum force of the first compression, ranged from 8.34 N for the control sample with shortening to 11.89 N for the variant with yellow beeswax oleogel. In the case of springiness, which determines the degree of crumb return to its initial height after the second compression, the most similar values were obtained by muffins with SH (1.14) and with candelilla wax (1.18). All variants of bakery goods were also similar in terms of cohesiveness.

Chewiness (CH) was also determined, which was defined as the force needed to break up the product in such a way that it could be swallowed [25]. It was found that for muffins containing different oleogels of rapeseed oil, it had a comparable value to the variant with shortening (Table 4).

### 2.5. Color Analysis of Gluten-Free Muffins

In order to determine the effect of the type of fat on the color of the crust of the baked goods, color analysis was performed using the reflection method. It showed significant differences between the parameters L*, a*, b*, the total color difference (∆E) between the variants containing oleogels and the control one with shortening (SH), and the browning index (BI) of muffin crust (Table 5).

The darkest crust among the tested gluten-free muffins was found in the control sample with shortening (Table 5). The value of the L* parameter was 69.87 and differed from the products containing oleogels. In the case of a* and b*, positive values were recorded in all variants (Table 5). Thus, the analyzed muffin crust was characterized by a warm shade, with the intensity of red (a*) from 7.99 (for the variant with sunflower wax oleogel) to 10.95 (for the muffin with candelilla wax oleogel) and yellow (b*) from 29.12 (for the variant with oleogel with beeswax yellow) to 32.46 (for SUN muffin variant) (Table 5). In the case of the ∆E parameter, which numerically shows the difference in total color between the crust of the control sample (SH) and muffins with oleogels, the lowest value (3.03) was found in the variant with sunflower wax. In the remaining bakery goods, significantly higher values of this parameter were recorded, and the maximum was 5.33 for the CAN variant (Table 5).

There were significant differences of BI parameter between three variants of muffins with beeswax yellow (64.66), white (70.70), or candelilla wax (74.87) oleogels (Table 5). The control sample did not differ significantly from the products with structured rapeseed oil.

### 2.6. Analysis of Gluten-Free Muffin Structure

**Figure 3:**
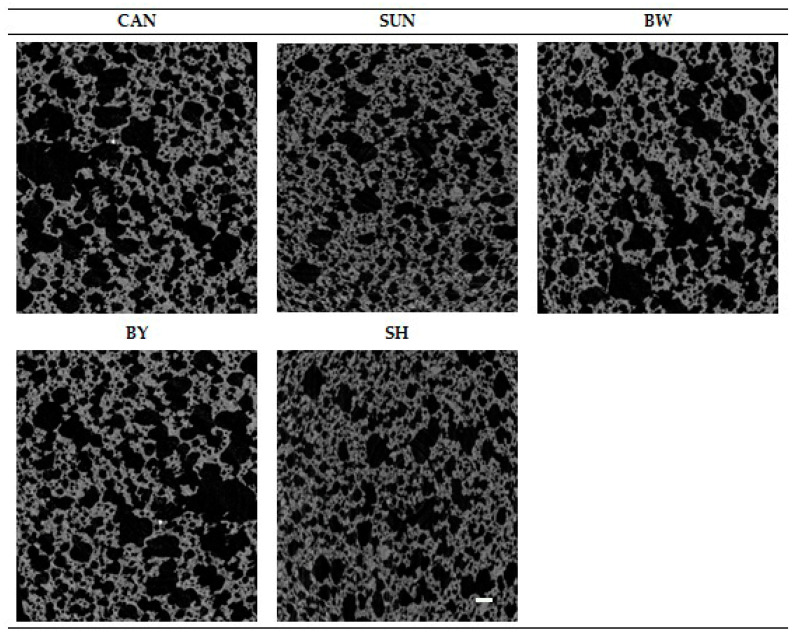
Two-dimensional (2D) reconstruction images of crumb of gluten-free muffins, containing shortening (SH) or oleogel with candelilla wax (CAN), sunflower wax (SUN), beeswax white (BW), or beeswax yellow (BY) as the fat component. Scale bar: 1 mm.

The microtomographic analysis of the structure of gluten-free muffins indicated differences between the variants (Table 6; Figure 3). Products containing oleogels with beeswaxes (white, yellow) or candelilla wax were characterized by the largest share of pores in the sample volume (over 50%), which is confirmed by 2D reconstruction images of their crumb (Figure 3). 

### 2.7. Fatty Acids Composition of the Lipid Fraction Extracted from Gluten-Free Muffins

The replacement of shortening (SH) with rapeseed oil oleogels resulted in a decrease of nutritionally undesirable saturated fatty acids (SFA) in the lipid fractions by approximately 40%, an increase in monounsaturated fatty acids (MUFA) by approximately 30%, and an increase in polyunsaturated fatty acids (PUFA) by approximately 15%. In all variants, no *trans* fatty acids isomers were found (Table 7).

In the samples containing oleogels, oleic acid (18: 1 cis-9) had the highest percentage (over 60%) of all fatty acids. This is due to the 95% rapeseed oil content, which (as mentioned earlier) is dominated by C18: 1 cis-9 [22]. Among saturated fatty acids, the highest percentage of palmitic acid 16:0 was demonstrated, which in the case of lipid fractions extracted from muffins with oleogels did not exceed 7%, while in the sample with control fat, its content was six times higher—over 43% (Table 7).

The results of the fatty acid profile of gluten-free muffins directly influenced the value of the atherosclerotic index (Table 7). On average, the values of this index were over three times lower for muffins with various types of oleogels compared to the variant with control fat—shortening. This proves a more favorable nutritional profile of fatty acids (FA) of new products with oleogels, among which variants with candelilla wax or beeswax white obtained the best relationship between the sum of the major SFA and the main classes of unsaturated FA. However, this hypothesis requires confirmation in further research. 

### 2.8. Quality of Fats Extracted from Muffins

**Figure 4:**
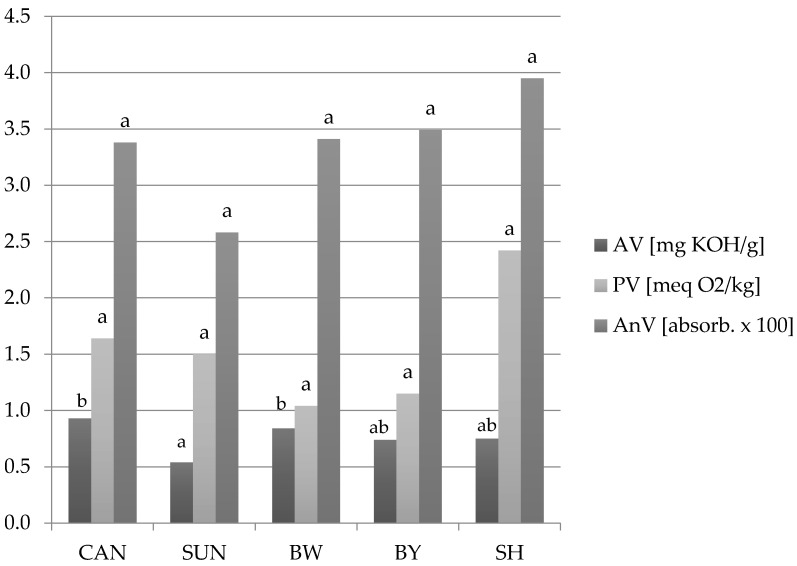
Acid (AV), peroxide (PV), and anisidine value (AnV) of lipid fractions extracted from gluten-free muffins with shortening (SH) or oleogel with: CAN—candelilla wax; SUN—sunflower wax; BW—beeswax white; BY—beeswax yellow; a, b—different letters above each type of column indicate statistical difference at α = 0.05.

To assess changes in the quality of fats during baking, the following factors were identified: acid, peroxide, and anisidine values in fresh shortening and rapeseed oil (Table 8), and in lipid fractions extracted from gluten-free muffins (Figure 4). The results indicate changes in the content of free fatty acids (referred to as AV), peroxide, and hydroxide (PV), as well as carbonyl compounds (AnV) due to temperature and baking time.

The acid value was increased in all lipid fractions comparing to fresh fats—from 0.37 for fresh shortening to 0.75 mg KOH/g for the lipid fraction extracted from muffin with SH, and from 0.25 for rapeseed oil to 0.93 mg KOH/g for post-extraction lipid with candelilla wax (Figure 4). The differences in AV between extracted fats may be due to the amount of free fatty acids contained in the structure-forming substances. This is confirmed by the AV declared by wax producers (Table 1). The lowest possible content of free fatty acids was characteristic for sunflower wax, which could result in obtained acid values by the lipid fraction of the muffin with SUN oleogel—0.54 mg KOH/g.

Fats extracted from muffins did not differ significantly in terms of peroxide values, which did not exceed 2.5 meq O_2_/kg fat. There were also no significant differences between the anisidine values, which ranged from 2.58 for the SUN lipid fraction to 3.95 for the SH variant (Figure 4). 

## 3. Discussion

### 3.1. Physical Properties of Raw and Baked Dough 

Saturated fatty acids (SFA) and *trans* fatty acids isomers (TFA) are responsible for the solid consistency of lipids, in connection with high content of solid phase and melting point [26]. According to Devi and Khatkar [27], shortenings (SH), because of the high amount of SFA, can keep air in their structure, which promotes proper aeration of the dough. The specific gravity (SG) reflects the degree of this process [28], which is caused by mixing or whipping ingredients. This study confirmed that the lower the SG value, the higher the content of air bubbles in the dough and the height of the baked muffin (Figure 1; Table 3). According to Ghotra et al. [29], good shortening’s aeration is caused by breaking the continuity of protein (*p*) and starch (s) chains and also by coating the surface of these molecules with a layer of fat. As a result, *p* and s do not adhere closely to each other, which in turn improves the aeration of the dough (Figure 2(1)), reducing the value of the specific gravity parameter (Figure 1).

In gluten-free products, water is an important factor involved in the starch gelatinization process, which leads to obtaining the appropriate volume and structure of final pastries [30]. The water content in these kinds of products should be between 15 and 30% [31], and in the analyzed gluten-free muffins, it was in this range: from 22.69% for the variant with shortening to 23.97% for the product with CAN oleogel (Table 2). Onacik-Gür et al. [32] noted 23.31% moisture content in traditional muffins, containing shortening (SH) and wheat flour. In our research, the control product (also with SH) but with a different type of flour—rice flour—obtained 23.25% of the above-mentioned parameter (Table 2). However, these values are similar. Therefore, it can be assumed that all variants of gluten-free muffins, including those with rapeseed oil oleogels, were characterized by alike water content to traditional products.

Texture profile analysis indicated that all tested products did not differ significantly in terms of hardness, springiness, cohesiveness, and chewiness (Table 4). In case of cohesiveness, comparable values of this parameter (from about 0.70 to 0.75) were obtained by Hesso et al. [33] for gluten cakes, containing a mixture of RO (70%) and anhydrous milk fat (30%). The researchers also found that the measurements of the cohesiveness of the crumb did not show any significant differences between tested variants similar to the gluten-free muffins (Table 4).

When a cake is baked, there are numerous physical, chemical, and biochemical changes, including color due to the Maillard reaction and the caramelization of sugars at high temperatures [34]. These processes are commonly known as browning, and it is a phenomenon related not only to visual but also nutritional aspects (e.g., it causes a decrease in the protein efficiency ratio) [35]. Ureta et al. [36] stated that the browning index (BI) can be used as an indicator of the degree of browning of sweet baked goods. Researchers obtained BI above 70 for sponge cake with wheat flour after baking 4 min longer with forced convection. The difference in baking time may be due to the 5 °C lower temperature (160 °C) compared to gluten-free muffins (165 °C), which influenced the browning index. On the other hand, the total color difference parameter (∆E) made it possible to record the similarity of the muffin crust to the control variant with shortening (Table 5). The results prove that the greatest sameness to the product with traditional solid fat in terms of color had a product with oleogel with sunflower wax (∆E 3.03). According to Tomasevic et al. [37], when the total color differences values are greater than 6, they are significant and well noticeable by the inexperienced observer. Therefore, it can be concluded that consumers would not find a meaning difference in the color of the crust of gluten-free muffins with shortenings or oleogels (∆E < 6 for all variants; Table 5). However, this thesis requires confirmation by means of a sensory analysis in further research.

The microtomography in food analysis enables better understanding of the product structure and thus the related physicochemical or sensory properties [38]. Total porosity (TP), which is one of the distinguishing features that describe the structure of the crumb, is defined as the ratio of the total number of pores to the volume of the sample. The gluten-free muffins differed in terms of this parameter (Table 6). Despite the best aeration of the control dough (Figure 2(1)), which was marked also as specific gravity (Figure 1), the number of pores in the crumb of this variant did not exceed 50% (Table 6). Therefore, it can be assumed that the tested shortening did not retain the air after mixing [27]. Lim et al. [16] for the control sample of muffins prepared with wheat flour obtained 78.31% of a total porosity. The reason for about 30% more pores in this variant, compared to gluten-free product with shortening, may be the type of flour used, which, through the content of gluten proteins, contributes to the formation of a network and retains gases during baking. However, this thesis requires confirmation in further research.

### 3.2. Profile of Fatty Acids and Chemical Quality of the Lipid Fraction

On the basis of the fatty acid composition of post-extraction lipid fractions, it was found that oleogels caused an increase in the proportion of polyunsaturated fatty acids, i.e., an almost two-fold increase in linoleic (18:2) and linolenic (18:3) compared to the control product. Lim et al. [16] also showed more than two-fold: an increase in all UFA and a decrease in SFA in fatty acid composition when oleogels were used in the muffin recipe instead of shortening. The results of this study as well as the values of the atherosclerotic index (Table 7) confirm the possibility of obtaining bakery products with increased nutritional value. 

As mentioned earlier, during the baking of raw cakes, under the influence of high temperature, numerous changes occur in it and the mutual interaction of its components. According to Żbikowska and Kowalska [39], baking may cause an increase in free fatty acids in cakes (referred to as Acid Value), as well as a significant increase in primary oxidation products, i.e., peroxides and hydroperoxides (PV). There is currently no standard for the quality of lipids after baking. However, there are known acid and peroxide value limits for fresh confectionery and bakery fats [40]: for AV, it is 0.5 mg KOH/g, and for PV, it is 3 meq O_2_/kg. They are more restrictive than the Codex Alimentarius requirements for refined vegetable oils (AV up to 0.6 mg KOH/g and PV up is 10 meq O_2_/kg) [22]. The European standards do not take into account the anisidine value, but Polish standards allow the maximum AnV of 8 (for refined vegetable oils) [41]. Fat raw materials, i.e., refined rapeseed oil used to make oleogels or shortening before baking, did not exceed all the above-mentioned limits (Table 8). Lipid fractions extracted from gluten-free muffins with oleogels or shortening obtained higher AV values than the acceptable limits for fresh confectionery and bakery fats: from 0.54 for fat with sunflower wax to 0.93 for fat with candelilla wax (Figure 4). However, considering the Codex Alimentarius standard for refined vegetable oils, the quality of the lipid fraction from SUN muffin variant is acceptable. 

The amount of peroxides and hydroperoxides formed did not differ significantly and did not exceed 2.5 meq O_2_/kg fat (Figure 4). The above-mentioned products of lipid oxidation under the influence of high temperature may degrade into carbonyl compounds (designated as AnV). It may result in a lower peroxide value (PV) [42] and higher anisidine value compared to the raw materials before baking. This thesis is confirmed by the AnV results for fresh fats and fractions, extracted from gluten-free muffins (Table 8; Figure 4). For post-extraction lipids, the value of this parameter ranged from 2.58 (for the sample from muffin with SUN oleogel) to 3.95 (for the probe with shortening). Taking into account the AnV results of fresh rapeseed oil (RO; 1.40) and shortening before baking (3.75), it can be concluded that in the wax-structured RO oxidation, products changed to a greater extent (Table 8; Figure 4), which is probably related to the oleogel preparation process (Section 4.1.2). However, statistical analysis did not show significant differences between the AnV of fats extracted from all variants of gluten-free muffins (Figure 4). Importantly, they did not exceed the allowed limit value—8 [41], which proves their acceptable quality in terms of degree of oxidative changes, even after baking. 

Possible further changes taking place in the lipid fraction of gluten-free muffins with oleogels, as well as those related to their texture, structure, and sensory quality, require further investigation.

## 4. Materials and Methods

### 4.1. Materials

The research material consisted of five variants of gluten-free muffins differing in the type of fat used, which was a shortening (SH) or one of the four variants of oleogels. These new solid lipids were prepared with various types of single gelling agents and waxes, at a constant concentration of 5% *w*/*w* in rapeseed oil (RO): candelilla, sunflower, and beeswax white or yellow. Their content in RO was determined in previous studies as an amount that ensures a constant consistency [43]. The following variants of bakery goods were made: CAN—muffins with oleogel with candelilla wax, SUN—with sunflower wax, BY—with yellow beeswax, BW—with white beeswax. The control sample of muffin contained shortening (SH).

In this work, rice flour was also used to produce gluten-free muffins. According to Matsuda [44], it is characterized by excellent nutritional value, functionality, and availability, and therefore, it may be one of the ingredients of food products for dietary intervention.

#### 4.1.1. Preparation of Gluten-Free Muffins

The recipe of bakery goods consisted of 100g of rice flour (Melvit Co., Olszewo Borki, Poland), 50 g of whole egg, 50 g of sugar (Krajowa Spółka Cukrowa, Toruń, Poland), 30 g of water, 20 g of fat-oleogel or shortening (Karlshamns, Sweden; saturated fatty acids + trans isomers (SFA and TFA) 47.9%, sum of monounsaturated fatty acid (Σ MUFA) 39.5%, sum of polyunsaturated fatty acids (Σ PUFA) 12.6%), 2 g of baking powder (Dr. Oetker Co., Gdańsk, Poland), and 0.75 g of salt (Solino Co., Inowrocław, Poland). Fat components (oleogel variant or shortening) were mixed with sugar using a mixer (Braun Multiquick; Braun GmbH, Kronberg im Taunus, Germany) for 1 min on speed turbo. After that, whole eggs were added and mixed for 3 min on speed turbo. Dry ingredients (rice flour, baking powder, and salt) were added and mixed at first on speed 2 for 1 min, and then for 4 min on speed turbo. The dough in the amount of 25 g was placed in molds rather than in a metal muffin pan. Raw muffins were baked in a convectional steam oven (UNOX, Cadoneghe, Italy) at 165 °C for 16 min. After that process, pastries were stored for 24 h in plastic bags at room temperature (20 ± 2 °C) until proper analysis. The baking was done in triplicate.

#### 4.1.2. Preparation of Oleogels

Plant waxes (Table 1): Candelilla (CAN) (Strahl & Pitsch Inc., West Babylon, NY, USA) or sunflower (SUN) (KahlWax, Trittau, Germany) and animal waxes: beeswax white (BW) or beeswax yellow (BY) (Strahl & Pitsch Inc., West Babylon, NY, USA) were used to prepare oleogels. The gelling agents were dissolved single in rapeseed oil (ZT Kruszwica S.A., Poland; SFA + TFA 7.12%, Σ MUFA 66.31%, Σ PUFA 26.08%) at a constant concentration of 5% by weight in a convectional steam oven (UNOX, Cadoneghe, Italy) at 80 °C to completely dissolve (15–25 min). After melting, all of the probes were poured into a plastic dish with a capacity of 50 cm^3^ and placed into a thermostatic cabinet (POL-EKO-APARATURA Sp. J., Wodzisław Śląski, Poland) at 20 °C for 24 h to solidify.

### 4.2. Methods

#### 4.2.1. Specific Gravity of Dough

The specific gravity of dough was made on the basis of the Martínez-Cervera et al. method [28]. The determination consisted in calculating the ratio of the mass of a standard vessel (ST) filled with the tested variant of the dough (W2) to the ST, which was filled with water (W1). The specific gravity of dough was calculated on the basis of the formula:

(1) $$\mathrm{ST}=\frac{W2}{W1}.$$

The determination was performed in triplicate.

#### 4.2.2. Pictures of the Microstructure of Raw Cakes

In order to observe the differences in the microstructure of the dough containing the addition of various types of fat (oleogels or shortening), the microscopic images were analyzed. A drop of samples was placed on a glass slide and covered with a coverslip. The study was performed in bright field and polarized light at 600× magnification using a biological microscope (Delta Optical, Mińsk Mazowiecki, Poland) and a microscope camera (Delta Optical DLT CAM PRO, Mińsk Mazowiecki, Poland) at room temperature (20 ± 2 °C).

#### 4.2.3. Geometric Dimensions, Weight, and Volume of Gluten-Free Muffins

The geometric dimensions (diameter and height) of the gluten-free muffins were determined using an electronic caliper (TCM, type: 234990, Tchibo, Hamburg, Germany). The height was set at the highest point of the product. The mass measurement was carried out on a technical scale (Radwag, Radom, Poland). All the above-mentioned determinations were performed eighteen times for each variant. Moreover, the volume of products using the rapeseed displacement method was determined [20]. The final result was the average volume of one muffin from a single variant in the cm^3^ unit.

#### 4.2.4. Texture Profile Analysis (TPA) of Crumb of Muffins

The texture profile analysis was carried out with the TX.AT plus device (Godalming, UK). It consisted in a double uniaxial compression of cube-shaped samples with a side of 20 mm in order to determine the textural properties of muffins: hardness, springiness, cohesiveness, and chewiness. The P/36R cylinder-shaped tip (36 mm in diameter) was used for the analysis. The compression was done to 75% of the initial height of the cubes, and the travel speed of the working part of the device was 5 mm/s. The analysis was performed in triplicate for each variant.

#### 4.2.5. Water Content of Gluten-Free Muffins

The water content test of crumb of bakery goods was carried out using a moisture analyzer (Radwag, Radom, Poland) for the program assigned to biscuit products. The test sample, weighing 1.000 ± 0.005 g, was placed in an aluminum pan with a diameter of 9 cm. The temperature of the drying process was in the range of 134–135 °C. The duration of the measurement was about 6 min. The result was given in percent. The assay was performed in triplicate for each variant.

#### 4.2.6. Color of the Crust of Baked Goods

The color determination of the crust of the baked muffins was performed with a Konica Minolta CR—200 colorimeter (Japan) in the CIE L*a*b* measurement system. The device was calibrated with a standard white plate, and the measurement diameter was 0.8 cm. The color of the crust of gluten-free muffins was determined applying: illuminant D65 and standard observer 2°. The number of readings per sample was six. In the measurement system, the L* coordinate relates to the brightness of the surface (0–100 scale), and a* relates to the color saturation, where positive values correspond to red, and negative values refer to green. For the b* parameter, positive values indicate yellowness, and negative values indicate blueness. Based on the L*, a*, and b* parameters, the browning index (BI) was determined [36]:

(2) $$\mathrm{BI}=\frac{\left[100\cdot \left(x-0.31\right)\right]}{0.172},$$

(3) $$x=\frac{\left(a^{*}+1.75\cdot L^{*}\right)}{\left(5.645\cdot L^{*}+{\;a}^{*}-3.012\cdot b^{*}\right)}.$$

The color coordinates were also used to determine the total color difference (∆E) between the crust of bakery goods. The variant of muffin that contained traditional solid fat (shortening) was used as a control sample. This parameter was calculated based on the following formula [37]:

(4) $$∆E=\sqrt{{\left(a_{p}^{*}-\;a_{x}^{*}\right)}^{2}+{\left(b_{p}^{*}-\;b_{x}^{*}\right)}^{2}+\;{\left(L_{p}^{*}-L_{x}^{*}\right)}^{2}}$$

where:

$a_{p}^{*}$, $b_{p}^{*}$, $L_{p}^{*}$—color coordinates of a muffin variant with oleogel;
$a_{x}^{*}$, $b_{x}^{*}$, $L_{x}^{*}$—color coordinates obtained for the control variant.

#### 4.2.7. Microtomographic Analysis of Muffins

The muffin structure was analyzed using a microtomograph SkyScan 1272 micro-CT (Bruckermicro CT, Kontich, Belgium) operated at the voltage of 40 kV. Cuboid crumb samples (10 mm × 10 mm × 15 mm) were rotated through an angle of approximately 52° to obtain images with a resolution 274 × 1024 pixels. The images were reconstructed to the 2D structure using NRecon 1.6.9.8. program and were analyzed in the CT Analyzer 1.13.11.0 + software. The total porosity (%) of the 2D structure was determined.

#### 4.2.8. Extraction of Lipid Fraction of Gluten-Free Muffins

Lipid fractions from all gluten-free muffins, i.e., those containing shortening or oleogels with candelilla wax, beeswax white, yellow or sunflower wax, were extracted at room temperature (20 ± 2 °C) with hexane (Avantor Performance Materials, Gliwice, Poland), which was then distilled in a BÜCHI laboratory evaporator B—491 (BÜCHI Labortechnik AG, Flawil, Switzerland) at a constant temperature of 60 °C. The chemical analyses were performed in triplicate.

##### Fatty Acids Profile and the Atherosclerotic Index

Shortening or oleogels lipid fractions were subjected to a methylation process. To two drops of fat, 2 cm^3^ of hexane were added; then, it was mixed in a Vortex centrifuge and then kept in a bath (AJL Electronic, Cracow, Poland) at 65 °C for about 10 seconds until completely dissolved. After cooling, 1 cm^3^ of a 0.5 M solution of KOH in methanol was added. After separation of the two layers, 500 μL of the upper (containing KT methyl esters) were taken into the vial, and then, 800 μL of hexane were added.

The fatty acid composition was determined in a TRACE 1300 gas chromatograph (Thermo Scientific, Waltham, USA) equipped with an FID detector and an autosampler. The separation of fatty acids (FA) methyl esters was carried out in a BPX 70 capillary column (60 m long and 0.22 mm in diameter). The starting temperature of the oven was 80 °C; after 2 min, it was linearly increased to 230 °C at a rate of 2.5 °C/min, and then, it was held for 6 min. The identification of the individual FA peaks was performed by comparing the retention time of the fatty acid methyl ester standards (FAME, Sigma-Aldrich, Saint Louis, MO, USA). Helium with a flow rate of 0.75 cm^3^/min was used as the carrier gas, and the injection size was 0.8 µL.

On the basis of the obtained fatty acid profiles, the atherosclerotic index (IA) was determined. It indicates a lipid health quality, and it is the ratio of the sum of the major SFA to the sum of the major unsaturated fatty acids. The atherosclerotic index was calculated according to the equation [45]:

(5) $$\mathrm{IA}=\frac{\left(4\cdot C14:0\;+\;C16:0\;+\;C18:0\right)}{\left(\Sigma \mathrm{MUFA}\;+\;\Sigma \mathrm{PUFA}-\omega -6\;+\;\Sigma \mathrm{PUFA}-\omega -3\right)},$$

where:

Σ—summatory, MUFA—monounsaturated fatty acids, PUFA—polyunsaturated fatty acids.

##### Quality Determination of Lipid Fraction

In order to determine the possibility of using oleogels, structured with waxes (candelilla, sunflower, beeswax white or yellow) as new shortening replacers, the quality of the muffin lipid fraction was analyzed 24 h after baking. Marked: acid value (AV), peroxide value (PV), and anisidine value (AnV) of the extracted lipid fraction based on ISO 660: 2010 (AV) [46], ISO 3960: 2012 (PV) [47], and PN-93/A-86926 (AnV) [48].

#### 4.2.9. Statistical Analysis

The statistical analysis was performed using the Statistica 13 program. The obtained results were analyzed using the one-way ANOVA of variance and the correlation matrix at the significance level α = 0.05.

## 5. Conclusions

Muffins containing rapeseed oil (OR) structured with waxes (candelilla, sunflower, beeswax yellow or white) obtained similar physicochemical properties to the control product with shortening. In terms of technology, all variants of oleogels made it possible to get products with good characteristics and increased nutritional value. The type of fat influenced the aeration of the raw cakes, which in turn affected the height of the finished products. The baking conditions, i.e., the temperature of 165 °C and the time of 16 min, caused the darkening of the control variant to the greatest extent. Temperature and baking time also influenced the quality of the lipid fraction. The results confirmed that shortening and sunflower-wax-structured rapeseed oil (SUN) underwent the least degree of hydrolytic and oxidative changes among the examined extracted fats. Its crust was also characterized by the most similar color scheme to the control variant. Therefore, it can be assumed that SUN oleogel could be the most promising alternative to professional shortening in the recipe of gluten-free products among the tested OR oleogels. However, this statement requires confirmation in further studies concerning, for example, the sensory quality of the baked goods.

## Figures and Tables

**Table 1:** Specification of the waxes used in the production of oleogels (manufacturer’s information).

Parameter	Type of Wax			
	Candelilla	Sunflower	Beeswax White	Beeswax Yellow
Acid value [mg KOH/g]	12–22	2–8	17–24	
Ester value [mg/g]	-	67–93	70–80	
Melting temperature [°C]	68.5–72.5	74–80	62–65	
Color	Yellow	Pale-yellowish	Off-White	Yellow

**Table 2:** Weight, geometric dimensions, volume, and water content of a muffin.

Variant of Muffin	Parameter				
	Weight [g]	Diameter [mm]	Height [mm]	Volume [cm^3^]	Water Content [%]
CAN	25.97 ± 0.16 a	50.53 ± 0.41 a	32.47 ± 0.44 a	53.70 ± 2.78 a	25.60 + 1.59 a
SUN	25.69 ± 0.06 a	51.27 ± 0.54 a	32.44 ± 0.53 a	54.81 ± 3.35 a	23.48 + 0.46 a
BW	25.78 ± 0.30 a	51.33 ± 025 a	32.50 ± 0.82 a	55.44 ± 4.22 a	23.25 + 0.38 a
BY	25.74 ± 0.12 a	51.24 ± 0.33 a	32.49 ± 0.46 a	55.55 ± 3.89 a	24.49 + 0.76 a
SH	25.70 ± 0.05 a	51.54 ± 0.79 a	33.18 ± 0.17 a	53.00 ± 0.91 a	23.25 + 0.38 a

a—homogeneous group.

**Table 3:** Correlation between the geometric parameters of baked muffins and the specific gravity of raw cakes.

	Weight *	Diameter *	Height (y)	Volume *
			r = −0.54	
Specific gravity (x)	-	-	R^2^ = 0.30	-
			Y = 45.53 − 10.97x	

* Statistically not different means at α = 0.05

**Table 4:** Hardness, springiness, cohesiveness, and chewiness of gluten-free muffins.

Variant of Muffin	Hardness [N]	Springiness [–]	Cohesiveness [–]	Chewiness [N]
CAN	8.94 ± 2.47 a	1.18 ± 0.36 a	0.76 ± 0.06 a	7.60 ± 2.01 a
SUN	11.88 ± 0.79 a	0.95 ± 0.02 a	0.73 ± 0.06 a	8.22 ± 0.21 a
BY	11.89 ± 1.20 a	0.97 ± 0.03 a	0.63 ± 0.06 a	7.13 ± 0.59 a
BW	9.75 ± 1.49 a	1.57 ± 0.55 a	0.68 ± 0.03 a	9.95 ± 3.85 a
SH	8.36 ± 1.06 a	1.14 ± 0.30 a	0.77 ± 0.08 a	7.49 ± 2.13 a

a—the same letter in each column indicates no statistical difference (α = 0.05).

**Table 5:** Color parameters of muffin crust.

Color Parameters					
Variant of Muffin	L*	a*	b*	∆E	BI
CAN	66.24 ± 0.06 a	10.95 ± 0.35 c	31.78 ± 0.56 bc	5.33 ± 0.92 b	74.87 ± 1.81 b
SUN	68.11 ± 0.37 b	7.99 ± 0.67 a	31.99 ± 0.27 bc	3.03 ± 0.42 a	69.48 ± 1.27 ab
BW	66.00 ± 0.56 a	9.82 ± 0.21 bc	30.55 ± 0.07 ab	5.15 ± 0.55 b	70.70 ± 0.72 b
BY	67.13 ± 0.21 ab	8.95 ± 0.01 ab	29.12 ± 1.36 a	4.69 ± 0.65 b	64.66 ± 3.33 a
SH	69.87 ± 1.19 c	9.11 ± 0.66 ab	32.46 ± 0.21 c	-	69.62 ± 2.66 ab

a, b, c—different letters in each column indicate significant differences (*p* ≤ 0.05).

**Table 6:** Total porosity of muffins crumb [%].

Variant of Muffin Crumb	Total Porosity [%]
CAN	53.64 ± 0.69 cd
SUN	42.96 ± 0.00 a
BW	52.46 ± 0.00 c
BY	55.07 ± 0.79 d
SH	49.49 ± 0.10 b

a, b, c, d—different letters in each column indicate significant differences (*p* ≤ 0.05).

**Table 7:** Fatty acid composition [%] and the atherosclerotic index (IA).

	CAN *	SUN *	BW *	BY *	SH *
C12:0	-	-	-	-	0.71
C14:0	-	-	-	-	1.07
C16:0	6.46	6.20	6.66	6.38	43.31
C18:0	1.75	2.05	2.32	2.38	5.11
C18:1 (trans-9)	-	-	-	-	-
C18:1 (cis-9)	62.32	63.15	62.98	61.22	37.88
C18:2 (cis-9,12)	19.34	20.20	18.94	19.29	10.50
C18:3 (cis-9,12,15)	6.58	6.52	6.16	7.32	0.70
C20:0	0.54	0.53	0.53	0.31	0.41
C20:1	1.19	0.88	1.26	1.17	-
C22:0	0.37	0.48	0.27	0.49	-
Others (<0,3)	1.45	-	0.88	1.44	0.31
Σ TFA	-	-	-	-	-
Σ SFA	9.12	9.26	9.78	9.56	50.61
Σ MUFA	63.51	64.03	64.24	62.39	37.88
Σ PUFA	25.92	26.72	25.10	26.61	11.20
ΣPUFA-ω-6	19.34	20.20	18.94	19.29	10.50
ΣPUFA-ω-3	6.58	6.52	6.16	7.32	0.70
IA	0.09	0.31	0.10	0.33	1.07

* Lipid fraction extracted from muffins contained SH—shortening or oleogels with: CAN—candelilla wax, SUN—sunflower wax, BW—beeswax white, BY—beeswax yellow.

**Table 8:** Quality parameters of fresh fats.

	AV [mg KOH/g]	PV [meq O_2_/kg]	AnV [absorb.·100]
Rafined rapeseed oil	0.25 ± 0.01	2.50 ± 0.52	1.40 ± 0.14
Shortening	0.37 ± 0.06	2.68 ± 0.08	3.75 ± 0.55

## Data Availability

Data is contained within the article.
